# Topological relationships between perivascular spaces and progression of white matter hyperintensities: A pilot study in a sample of the Lothian Birth Cohort 1936

**DOI:** 10.3389/fneur.2022.889884

**Published:** 2022-08-24

**Authors:** Abbie Barnes, Lucia Ballerini, Maria del C. Valdés Hernández, Francesca M. Chappell, Susana Muñoz Maniega, Rozanna Meijboom, Ellen V. Backhouse, Michael S. Stringer, Roberto Duarte Coello, Rosalind Brown, Mark E. Bastin, Simon R. Cox, Ian J. Deary, Joanna M. Wardlaw

**Affiliations:** ^1^College of Medicine and Veterinary Medicine, University of Edinburgh, Edinburgh, United Kingdom; ^2^Department of Neuroimaging Sciences, Centre for Clinical Brain Sciences, University of Edinburgh, Edinburgh, United Kingdom; ^3^Department of Psychology, University of Edinburgh, Edinburgh, United Kingdom

**Keywords:** perivascular spaces, Virchow-Robin Spaces, white matter hyperintensities, aging, longitudinal, MRI, brain, small vessel disease

## Abstract

Enlarged perivascular spaces (PVS) and white matter hyperintensities (WMH) are features of cerebral small vessel disease which can be seen in brain magnetic resonance imaging (MRI). Given the associations and proposed mechanistic link between PVS and WMH, they are hypothesized to also have topological proximity. However, this and the influence of their spatial proximity on WMH progression are unknown. We analyzed longitudinal MRI data from 29 out of 32 participants (mean age at baseline = 71.9 years) in a longitudinal study of cognitive aging, from three waves of data collection at 3-year intervals, alongside semi-automatic segmentation masks for PVS and WMH, to assess relationships. The majority of deep WMH clusters were found adjacent to or enclosing PVS (waves−1: 77%; 2: 76%; 3: 69%), especially in frontal, parietal, and temporal regions. Of the WMH clusters in the deep white matter that increased between waves, most increased around PVS (waves−1–2: 73%; 2–3: 72%). Formal statistical comparisons of severity of each of these two SVD markers yielded no associations between deep WMH progression and PVS proximity. These findings may suggest some deep WMH clusters may form and grow around PVS, possibly reflecting the consequences of impaired interstitial fluid drainage *via* PVS. The utility of these relationships as predictors of WMH progression remains unclear.

## Introduction

Enlarged perivascular spaces (PVS) and white matter hyperintensities (WMH) are two of the most common neuroradiological signatures of cerebral small vessel disease (cSVD) (1) in older age, a condition resulting from pathological processes affecting the small arteries, veins, and capillaries in the brain (2). cSVD is identified by the coexistence of microhaemorrhages, WMH, and/or lacunes/lacunar infarcts, usually accompanied by enlarged PVS, in brain magnetic resonance imaging (MRI) scans. PVS are fluid-filled spaces that surround small penetrating blood vessels (1), which provide a route for the clearance of brain waste products (3). When enlarged, they become visible in brain MRI with the appearance of small linear or round structures depending on how they are positioned (i.e., parallel or perpendicular) with respect to the imaging plane. The cause of PVS enlargement is not fully understood but is thought to be related to impaired fluid drainage (4), as a consequence of several pathological processes that affect the cerebral microvasculature, including blood–brain barrier dysfunction, vessel stiffening, and reduced vessel pulsatility (5, 6). MRI-visible PVS, although seen also in scans of healthy adults without cSVD, have been associated with age, vascular risk factors, especially hypertension (7, 8), and WMH (1). WMH, on the contrary, are considered neuropathological features and appear in a wide spectrum of disorders. They have traditionally been associated with de-/dysmyelination processes and axonal degeneration (9), but advances in MRI have revealed that their presence may also reflect changes in interstitial fluid flow and increased water content in the white matter, especially in the earlier stages of cSVD (10). Like PVS, our understanding of WMH pathogenesis is poor, but they are strongly associated with vascular risk factors (11, 12), other imaging features of cSVD (13), cognitive decline, gait disturbance, and an increased risk of stroke and dementia (14).

Although PVS and WMH are commonly seen together in brain MRI scans of cognitively normal older individuals, they are clinically silent, and only a few studies have analyzed their relationship with conflicting results. Thus far, these studies have based their analyses on PVS and WMH severity, as determined by visual rating scores, for which subjectivity may have contributed to the conflicting findings. For instance, two cross-sectional studies found an association between the severity of enlarged PVS with WMH in two different clinical groups: lacunar stroke patients (15) and community-dwelling septuagenarian individuals (16); yet a recent meta-analysis including these studies and six others found no statistically significant associations between the two imaging markers (8).

Computational methods have been developed to quantify a PVS burden from MRI scans, helping to overcome the subjectivity associated with these scoring systems [e.g., (17–22), for mentioning just a few]. A recent study found stronger positive associations between computationally derived PVS metrics and WMH severity than visual scores (23). These cross-sectional associations suggest that widening of PVS might reflect small vessel endothelial dysfunction and impaired interstitial fluid drainage that contributes to a greater WMH burden and accumulating brain damage in aging and cSVD (23). Longitudinal associations between these PVS metrics and WMH have not been studied. However, a high burden of PVS in the basal ganglia (determined by visual rating) has been associated with WMH progression after adjusting for age, sex at birth, and vascular risk factors (24).

There is a growing interest in topological relationships between PVS and WMH. Understanding how PVS and WMH are spatially related to one another in the brain may reveal important insights into their underlying mechanisms and formation. It has been observed that WMH appear to form around PVS in stroke patients (25), typically in the parietal and posterior and lateral temporal regions. A recent study (26) explored the topological associations between WMH and PVS in randomly selected WMH clusters identified in a cross-sectional sample of 136 adults without previous history of stroke, brain trauma, or any neurological or systemic disease and reported that most of the randomly selected deep WMH clusters analyzed were spatially connected to PVS. But longitudinal data are required to directly characterize the within-person temporal dynamics of the processes related to the evolution of WMH and PVS and their possible synergy. The findings from Huang et al. (26), if confirmed in a longitudinal and more heterogeneous sample in terms of vascular disease, would provide further evidence to support a mechanistic link between both cSVD features and reveal whether PVS could predict WMH progression. Being able to identify patients with WMH that are more likely to progress may help to prevent development of associated neurological symptoms and conditions through earlier clinical intervention (27). We hypothesize that in cognitively normal older adults, (1) more deep WMH clusters would probably be spatially close to PVS (than not close) and increase in size around them and (2) those WMH clusters close to PVS, with time, will increase in size more than the WMH clusters that are distant from PVS.

## Materials and methods

### Subjects and clinical data

We utilized brain MRI, clinical, and demographic data from a randomly selected sample of participants of the Lothian Birth Cohort 1936 Study (https://en.wikipedia.org/wiki/Lothian_birth-cohort_studies), a longitudinal study of cognitive aging comprising community-dwelling individuals from in and around Edinburgh born in 1936. All participants provided written consent to take part in the study under protocols approved by Lothian (REC 07/MRE00/58) and Scottish Multicentre (MREC/01/0/56) Research Ethics Committees. The methods used for MRI acquisition and clinical data in this cohort have been reported previously (28–30). Participants had their first brain MRI scan at the second wave of testing at a mean age of 72.6 years and subsequent MRI examinations spaced at 3-year intervals (30). For this study, we randomly (i.e., using a random number generator function in MATLAB R2019b) selected a sample of 32 participants with brain MRI available for the first three consecutive scanning waves (i.e., a total of 96 individual MRI scans) with high-quality image data. Vascular risk factors, which included presence or absence of hypertension, hypercholesterolaemia, diabetes mellitus, stroke, and history of cardiovascular disease, were self-reported at each wave (30).

### Image acquisition

All brain images were acquired at the Western General Hospital of Edinburgh in a GE Signa Horizon HDx 1.5-T clinical scanner (General Electric, Milwaukee, WI) following the research acquisition protocol described in Wardlaw et al. (29) and ensuring full-brain coverage. In brief, the 3D T1-weighted sequence (160 slices) had an acquisition matrix of 192 × 192 and a voxel size of 1 × 1 × 1.3 mm^3^. The axial T2-weighted fast spin echo sequence (TR/TE = 11,320/102 ms, 80 slices) was acquired with a 256 × 256 matrix and a voxel size of 1 × 1 × 2 mm^3^. The axial fluid-attenuated inversion recovery (FLAIR) fast spin echo sequence (TR/TE/TI = 9,000/140/2,200 ms, 40 slices) was acquired with a 256 × 192 matrix and a voxel size of 1 × 1 × 4 mm^3^. The axial T2^*^-weighted gradient echo sequence (TR/TE = 940/15 ms, 80 slices) was also acquired with a 256 × 192 matrix, but with a voxel size of 1 × 1 × 2 mm^3^. None of the sequences had an inter-slice gap.

### Image processing

Each study participant's image data were processed and checked individually. For each participant, all image sequences from all waves were co-registered to the T2-weighted image acquired in the first scanning wave using rigid-body registration (linear, six degrees of freedom) through FSL-FLIRT (31). We used existent binary masks of WMH from the three waves and PVS in the centrum semiovale from the first imaging wave generated as previously published (23, 32, 33) (Supplementary Figure 1). In brief, for the first scanning wave (i.e., referred hereby as wave 1) WMH masks were generated using a multispectral method that combines T2^*^-weighted and FLAIR images mapped in the red–green color space and quantised to facilitate a robust thresholding using minimum variance quantisation (32). For the other two waves, WMH binary masks were generated from a statistical analysis of the FLAIR-normalized intensities, seeking full compatibility with the semi-automatic approach applied in the first wave, and a higher level of automation: while in the multispectral approach the Gaussians that describe the intensity distribution of the tissue classes in the FLAIR image were adjusted by the observer aided by a combination with another T2-weighted-based image, in the other this adjustment was performed automatically. In brief, hyperintense voxels on FLAIR were identified by thresholding intensity values equal to 1.69^*^standard deviation above the mean, using an adjusted implementation from Zhan et al. (34). The resulting hyperintense areas unlikely to reflect pathology (i.e., artifacts and cortex) were removed automatically using a lesion distribution template generated from the segmentation results of the first wave. Further refinement was achieved by applying Gaussian smoothing, followed by the removal of voxels with intensity values below 0.1 and *z*-scores below 0.95. All WMH masks were checked and manually edited to ensure the segmentation was as accurate as possible. Bland–Altman analyses yielded a mean WMH volume difference (SD) of 0.38 (1.29) ml and an intra-class correlation coefficient of 0.938, in 15 randomly selected individual datasets that were segmented with both implementation approaches. Voxel-wise reliability analyses comparing the WMH masks manually edited after applying both approaches show only scattered differences in the periventricular boundaries and Dice similarity coefficient of 0.6 (SD = 0.128), similar to the published inter-observer differences using the same method (35). No clusters (i.e., of three or more voxels) of differences were identified.

Segmentation masks for PVS in the deep normal-appearing white matter (i.e., excluding the internal and external capsules that are part of the region clinically defined as basal ganglia for the purposes of PVS identification) were generated using a computational method described previously (23, 36), on T2-weighted images. Succinctly, images were resampled from 256 × 256 × 80 to 256 × 256 × 160, using spline interpolation, to perform the PVS segmentation in images with 1-mm^3^ isotropic voxels. According to Wardlaw et al. (1) and Valdés Hernández et al. (37), PVS were identified as tubular structures with lengths between 3 and 50 mm. Tubular structures in the “isotropic” T2-weighted images were enhanced using the Frangi filter in its 3D version, optimized for this purpose with the parameters described by Ballerini et al. (36), which, according to Ballerini et al. (23), enhances structures with widths above 0.5 and below 2.5 voxels. The output from the filter was thresholded, binarised, and quantified in the region of interest. We used 3D connected component analysis to computationally identify and assess the PVS. Segmentation masks for PVS inside WMH were generated using the same method (i.e., thresholding the output of the 3D Frangi filter) but in a fused image obtained by subtracting the FLAIR image from the T2-weighted image after both being corrected for bias-field inhomogeneities using FSL-FAST (38) and their intensities being normalized. This is to discern whether the low-intensity voxels within the WMH in FLAIR correspond to the PVS-like hyperintense structures identified using the T2-weighted image. Combining sequences to increase distinction of PVS-like structures is a well-established procedure (17, 21). The images were individually checked for noise and other artifacts that would affect accuracy of PVS segmentation. To avoid errors in misclassifying small lacunes resembling PVS as PVS, neuroradiological reports were checked.

### Visual assessments

**Figure 1:**
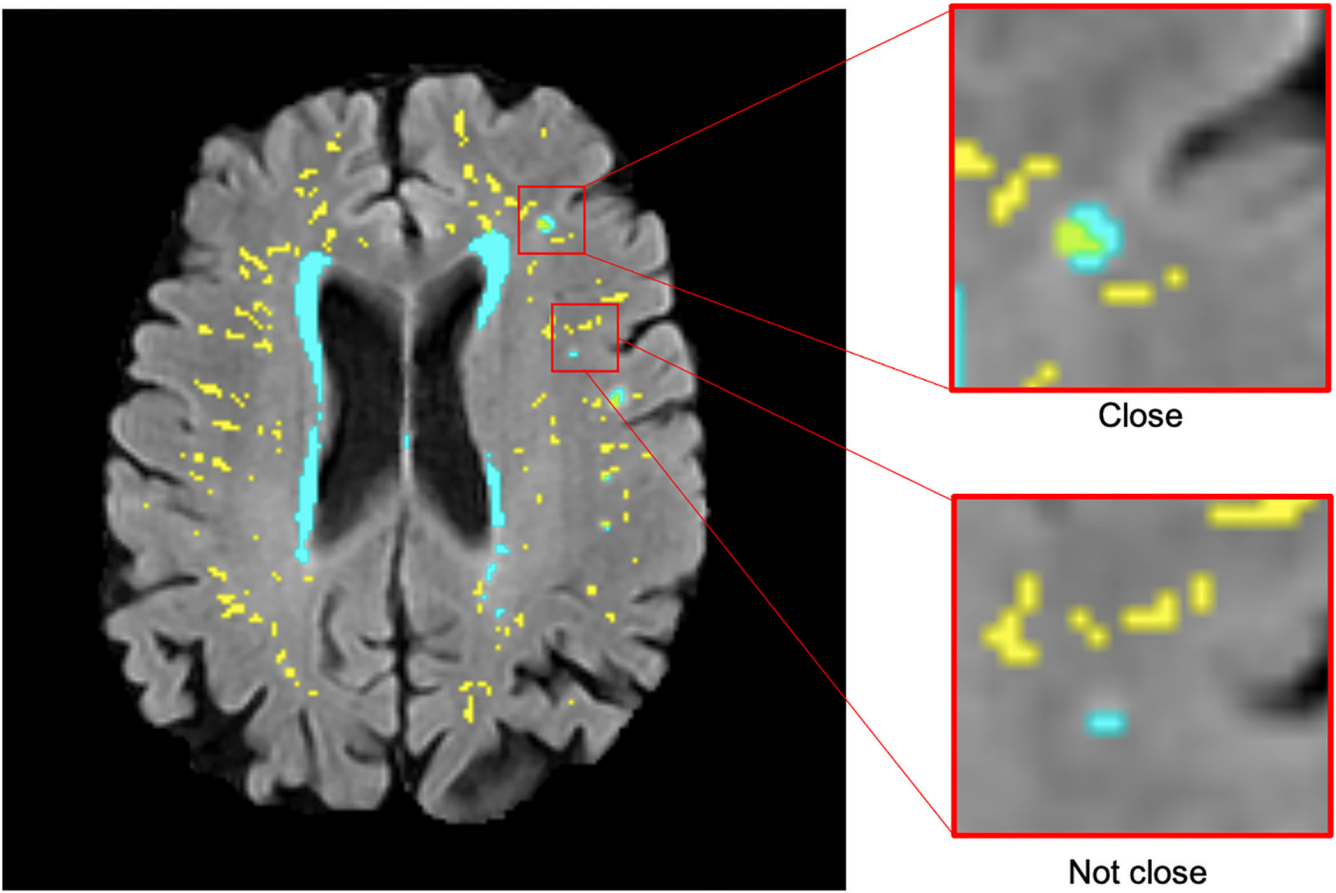
Examples of deep WMH in the baseline scan that would be classified as “close” and “not close” to baseline PVS when determining cross-sectional topological relationships (segmentation masks for WMH in cyan and PVS in yellow, overlaid on FLAIR MRI).

Adjacency/closeness (or not) of PVS with WMH clusters [minimum size 3 mm diameter according to Wardlaw et al. (1)] was recorded while/by visually inspecting all slices where PVS were segmented. Initially, the inspection was done in axial slices, and they were double-checked in all radiological orientations throughout the sample in all waves. All visual assessments, performed with MRIcron v1.0.20190902 (https://www.nitrc.org/projects/mricron/), were blinded to visual rating scores for PVS and WMH and participants' clinical and demographic information. These were repeated by the same observer to ensure a perfect intra-observer agreement [mean differences in PVS count ± 95% confidence interval are equal to −0.207 ± 1.637, intra-class correlation coefficient (ICC) 0.99917 for wave 1 and −0.034 ± 1.361, ICC = 0.99961 for wave 2, see Bland–Altman plot in Supplementary Figure 2]. Deep WMH clusters were defined as regions or voxel clusters of WMH not contiguous with the WMH located in the periventricular lining. Periventricular WMH caps surrounding the horns of the lateral ventricles were counted as deep WMH clusters if they extend more than 13 mm from the ventricular surface into the deep white matter (39). Cross-sectional topological relationships were assessed on the first scanning wave. Each deep WMH cluster was recorded and classified as either “close” or “not close” to a perivascular spaces (PVS) in the first scan in co-registered T2-weighted and FLAIR images after superimposing the PVS and WMH binary masks in different contrasting colors (Figure 1). Deep WMH were defined as “close” to a PVS if their segmentation was overlapping or contiguous with a PVS and “not close” if it was not overlapping or contiguous. Adjacent slices were carefully inspected to ensure not to miss or double-count any PVS. Each deep WMH cluster was also labeled, and the region of the brain it was found in was recorded—either frontal, parietal, temporal, or occipital, determined by examining lobar segmentations from a digital anatomical atlas (40).

**Figure 2:**
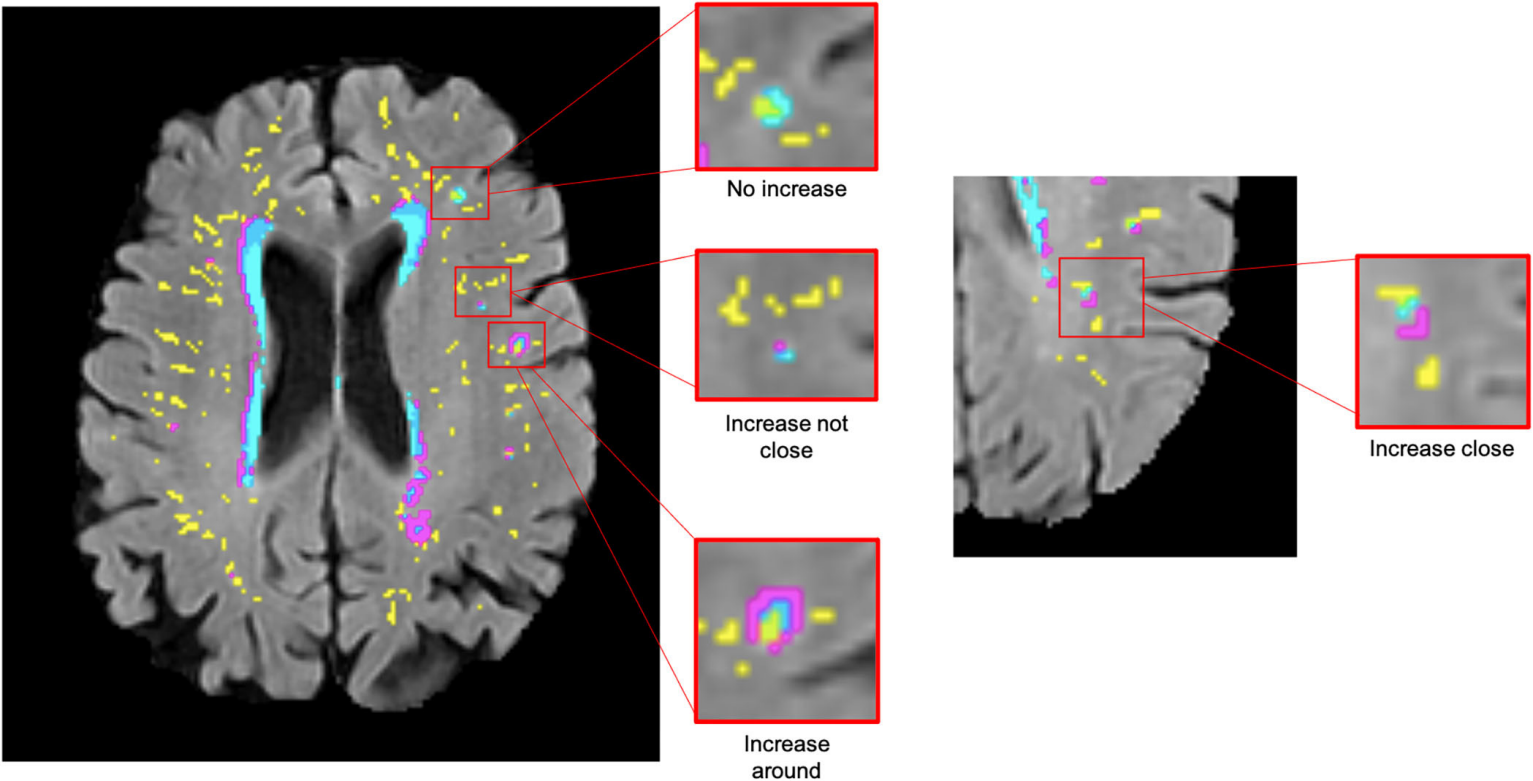
Examples of deep WMH for each of the different categories used to determine longitudinal topological relationships (segmentation masks for WMH in cyan, change in WMH in violet, and PVS in yellow, overlaid on FLAIR MRI).

To identify topological relationships longitudinally, the total number of deep WMH clusters was counted again. This time, each was classified according to how their morphology changed relative to baseline PVS between consecutive waves (e.g., waves 1 and 2 and waves 2 and 3) in each participant. A further mask that represented the change in WMH between waves was also overlaid in the FLAIR image to facilitate this assessment (Figure 2). These relationships were defined as follows: “increase around”—deep WMH cluster increased so that more voxels of the WMH mask became contiguous with a PVS; “increase close”—deep WMH cluster previously considered “close” to a PVS, increased in size, but no more of it became contiguous with a PVS (i.e., the adjacency between both—PVS and WMH—masks observed was owed to the same number of voxels as in the previous wave); “increase not close”—deep WMH cluster increased but it was still not contiguous with a PVS; and “no increase”—no visible change in the deep WMH cluster (Figure 2). To avoid WMH cluster change in size being influenced by differences in WMH segmentation algorithms, this was only considered if the change was perceived as nearly having doubled (or halved) or more the original cluster size. Again, all deep WMH clusters counted were recorded according to the lobar brain region.

### Statistical analysis

A statistical analysis was performed in R (version 3.6.2). First, the counted data per overall individual brain scan were summarized to show the median count and distribution within the sample, per category. Cross-sectional and longitudinal topological relationships were analyzed by calculating percentages per category, both overall and per brain region. To allow a longitudinal analysis of the cross-sectional relationships, a change in count of deep WMH clusters “close” and “not close” to baseline PVS between waves was simplified into a binary variable where “1” indicates an increase in the number of deep WMH clusters and “0” indicates otherwise (i.e., no change or a decrease). A binary logistic regression with random effects was used to model the association between the change in the number of deep WMH clusters and their location relative to a PVS. Random effects accounted for variation between participants and for correlation within participants, i.e., an individual could contribute more than one row of data to the analysis. Data per overall brain were used, and separate models were performed for waves 1–2 and waves 2–3. In addition to unadjusted models, we evaluated models that corrected for sex at birth and vascular risk factors, including hypertension, hypercholesterolaemia, diabetes mellitus, and history of cardiovascular disease, with a maximum of three predictors per model. We estimated our sample size guided by the only study that visually assessed the topological relationships between WMH and PVS (26), which randomly selected 600 deep WMH clusters from a cross-sectional sample for analysis. In our sample, at baseline (i.e., only in wave 1), the total number of deep WMH clusters counted surpasses 600. Resources available for this study allowed us to process data from 29 individuals. Nevertheless, given the exploratory nature of this study, the complexity, and nature of the assessments, to ensure reproducibility, comparability, trustworthiness, and objectivity in the analyses, this estimate was considered appropriate (41). We consider the primary value of this study to be an indication of feasibility. Moreover, although the LBC 1936 Study, which provided data for the current analysis, enrolls 728 individuals in its first MRI scanning wave, it is inappropriate to use own data for a sample size calculation for an analysis with the same data (41).

To further evaluate whether the spatial relationship between deep WMH clusters and PVS could have been (or not) influenced by the PVS burden, we analyzed the *R*^2^ value of the univariate linear regression models that had the number of PVS close to WMH clusters as independent variables and the WMH volume at each wave as a dependent variable, and we further compared these with the R^2^ values of the models that have, instead, the PVS volume and the average PVS size as dependent variables. As *R*^2^ measures the strength of the relationship between the set of independent variables and the dependent variable, if the spatial relationship between PVS and WMH is determined by the PVS burden we would expect higher *R*^2^ values (i.e., less variance around the fitted line) in the models that relate the PVS burden (i.e., total volume, mean PVS size) to the WMH volume. In addition, we calculated the density of PVS “close” and PVS “not close” to WMH at the three scanning waves and conducted a paired t-test (two-tailed) at each wave comparing the densities of PVS “close” and PVS “not close”. If our hypothesis on the existence of a spatial relationship between deep WMH clusters and PVS is true, the PVS density (i.e., number per ml) close to deep WMH clusters will be greater than (and significantly different from) the PVS density not close to WMH.

## Results

### Sample characteristics

Data from three participants were excluded due to inaccuracies in their PVS segmentations mainly due to the presence of motion artifacts in the T2-weighted images. One participant was not included in the models performed between waves 1 and 2 as no deep WMH clusters were counted in either of these waves. The mean age of the sample at baseline was 71.87 years (SD = 0.38). The follow-up MRI scans from waves 2 and 3 were obtained three and six years later, respectively (i.e., at a mean age ~75 and 78 years). Table 1 shows the baseline characteristics of the sample including sex at birth, vascular risk factors, and Fazekas visual rating scores of WMH.

**Table 1:** Baseline participant characteristics, *n* = 29 (CVD = cardiovascular disease).

	**Male/Female**
Gender	17/12 (58.62%/41.38%)
	**Deep/Periventricular**
WMH Fazekas scores (median [QR1 QR3])	1 [1 1]/1[1 2]
Diabetes	1 (3.45%)
Hypertension	14 (48.28%)
Hypercholesterolaemia	9 (31.03%)
Stroke	0 (0.00%)
History of CVD	9 (31.03%)

### Cross-sectional topological relationships

**Figure 3:**
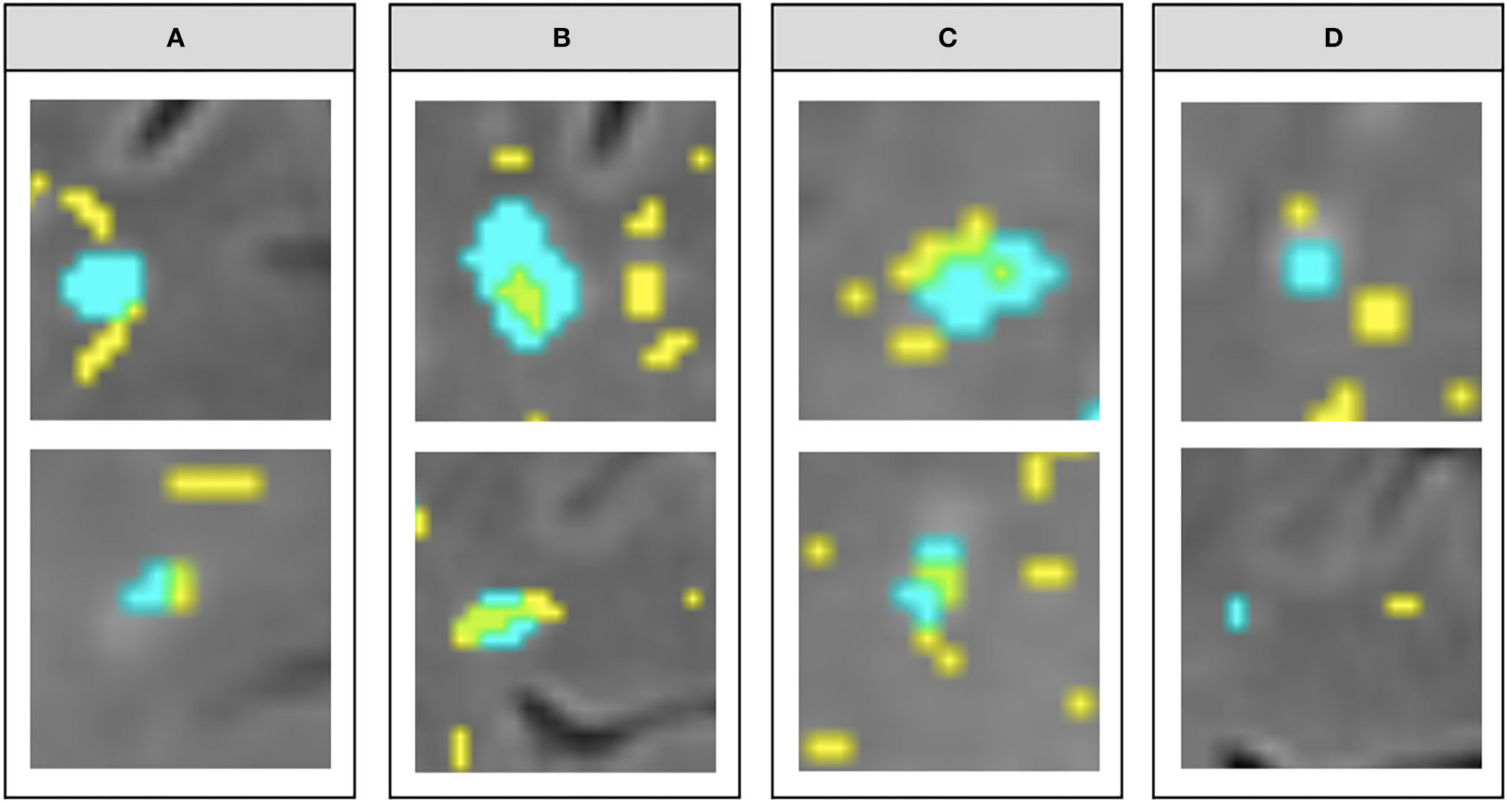
Examples of the different topological types observed within the “close” vs. “not close” classification used for describing cross-sectional topological relationships. **(A–C)** show variations within deep WMH classed as “close,” while **(D)** shows variations within those classed as “not close” (segmentation masks for WMH in cyan and PVS in yellow, overlaid on FLAIR MRI).

**Figure 4:**
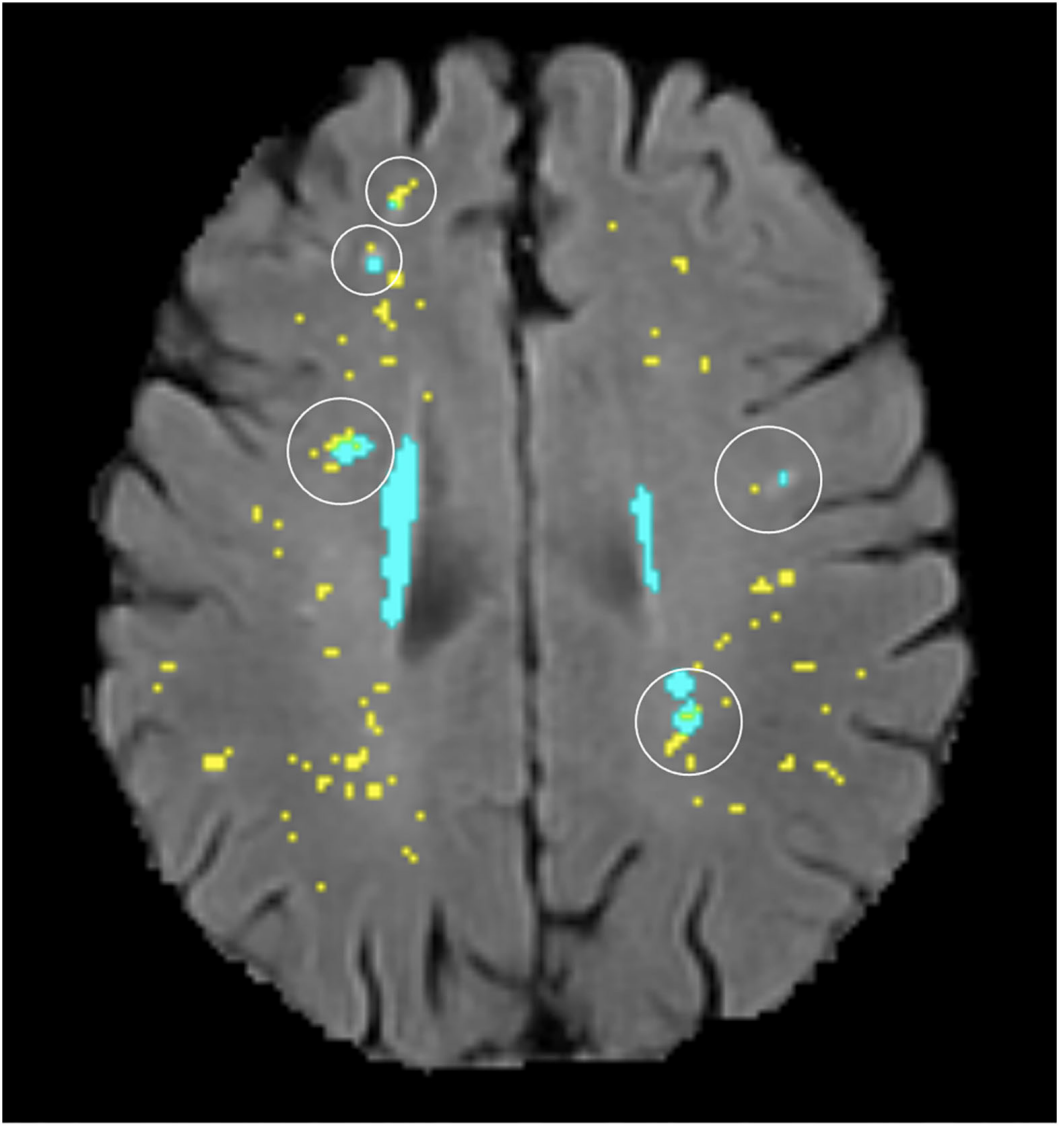
Illustration of several different topological types (circled) existing in the same participant (segmentation masks for WMH in cyan and PVS in yellow, overlaid on FLAIR MRI).

Figure 3 illustrates the variations in the cross-sectional topologies observed. For deep WMH clusters classed as “close” to a PVS, most bordered a PVS with a small area of overlap (Figure 3A). Within deep WMH clusters that were classed as “not close” to a PVS, there was considerable variability in the distance separating them (Figure 3D). Many of these different topologies existed simultaneously in the same participant (Figure 4).

**Figure 5:**
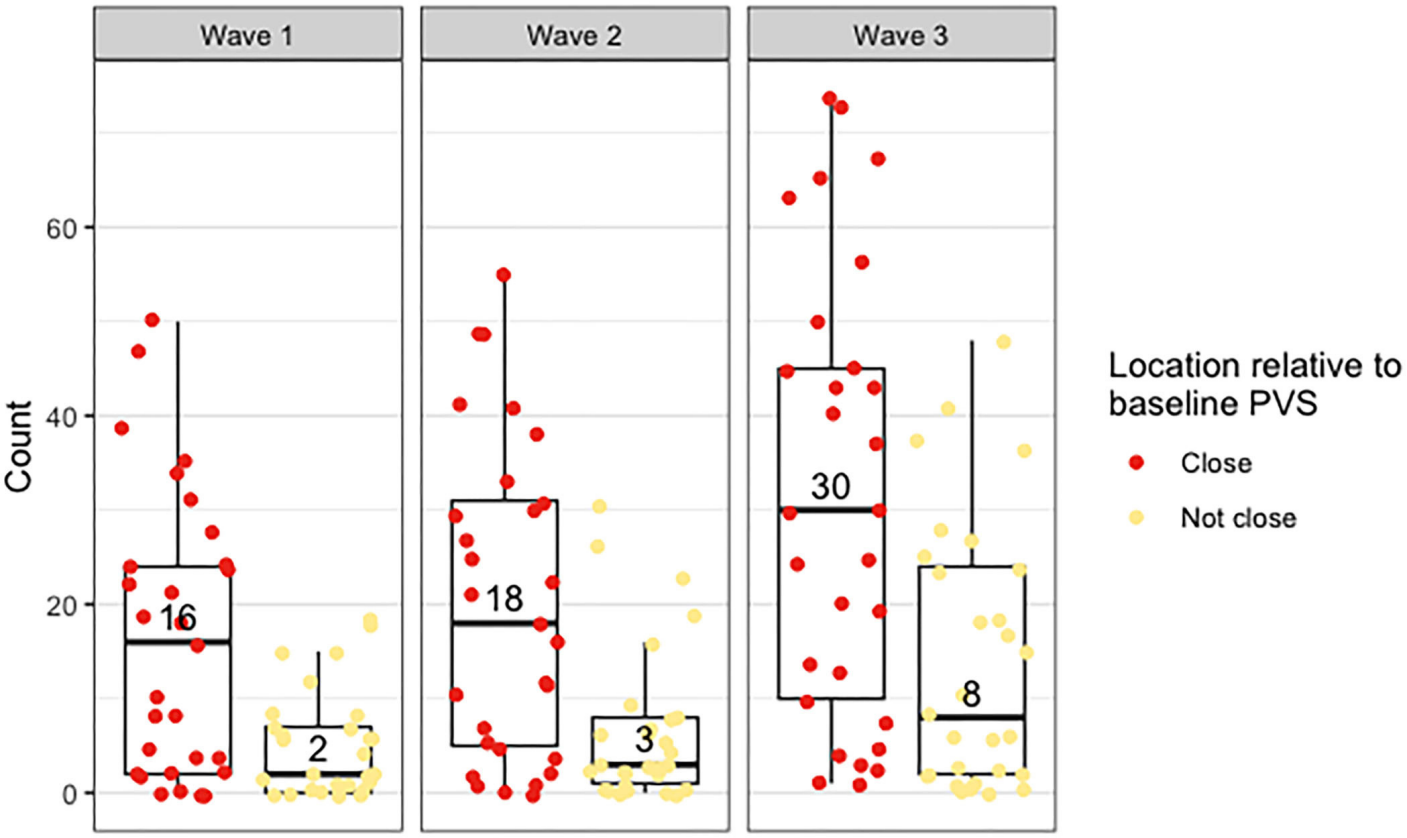
Distribution of counts of deep WMH in waves 1, 2, and 3, found close and not close to baseline (i.e., wave 1) PVS. The points plotted on each boxplot represent a different participant in the sample, and the numbers inside the boxplots correspond to the median count.

**Figure 6:**
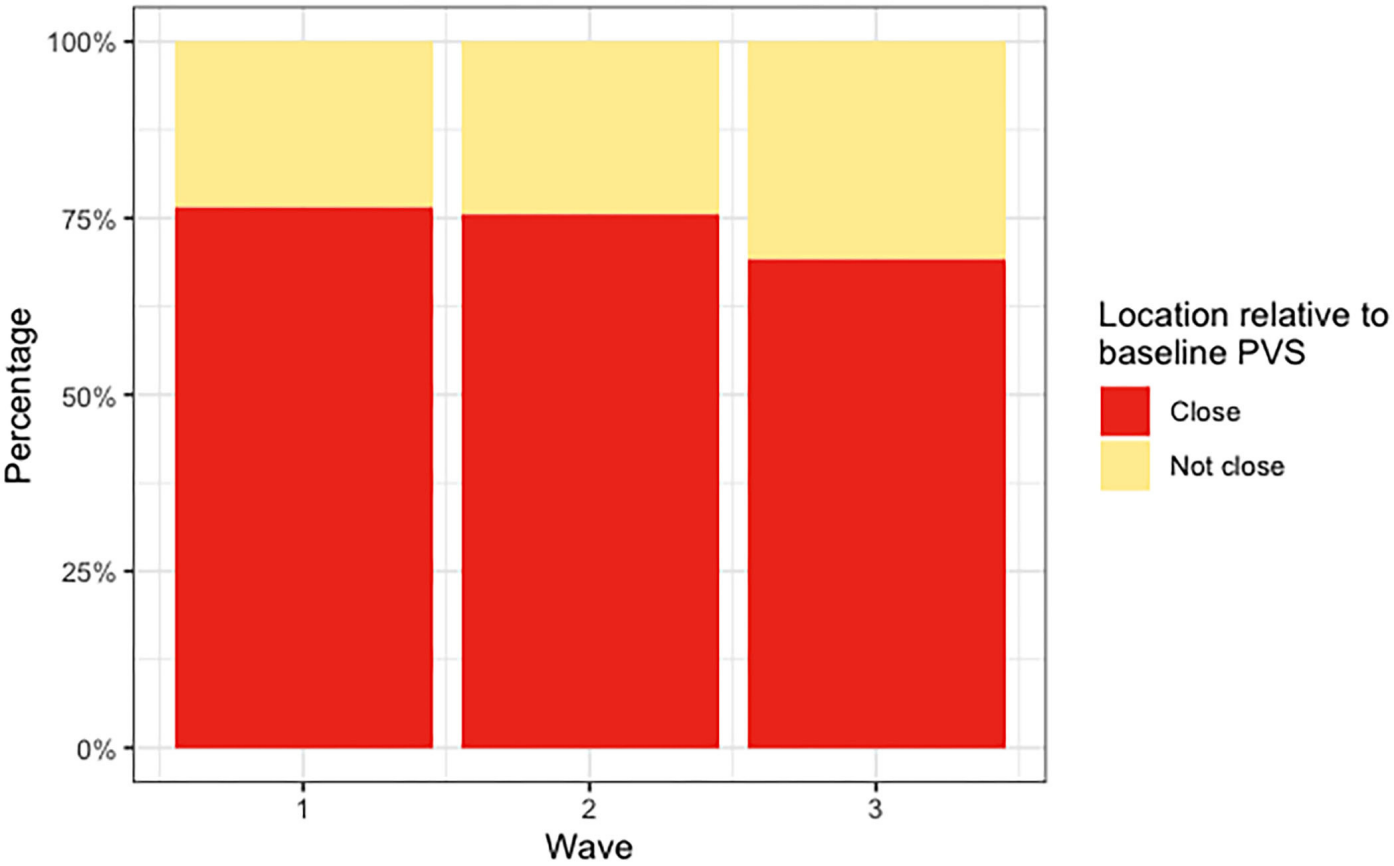
Percentages of deep WMH in waves 1, 2, and 3 that were found “close” and “not close” to baseline (i.e., wave 1) PVS.

**Figure 7:**
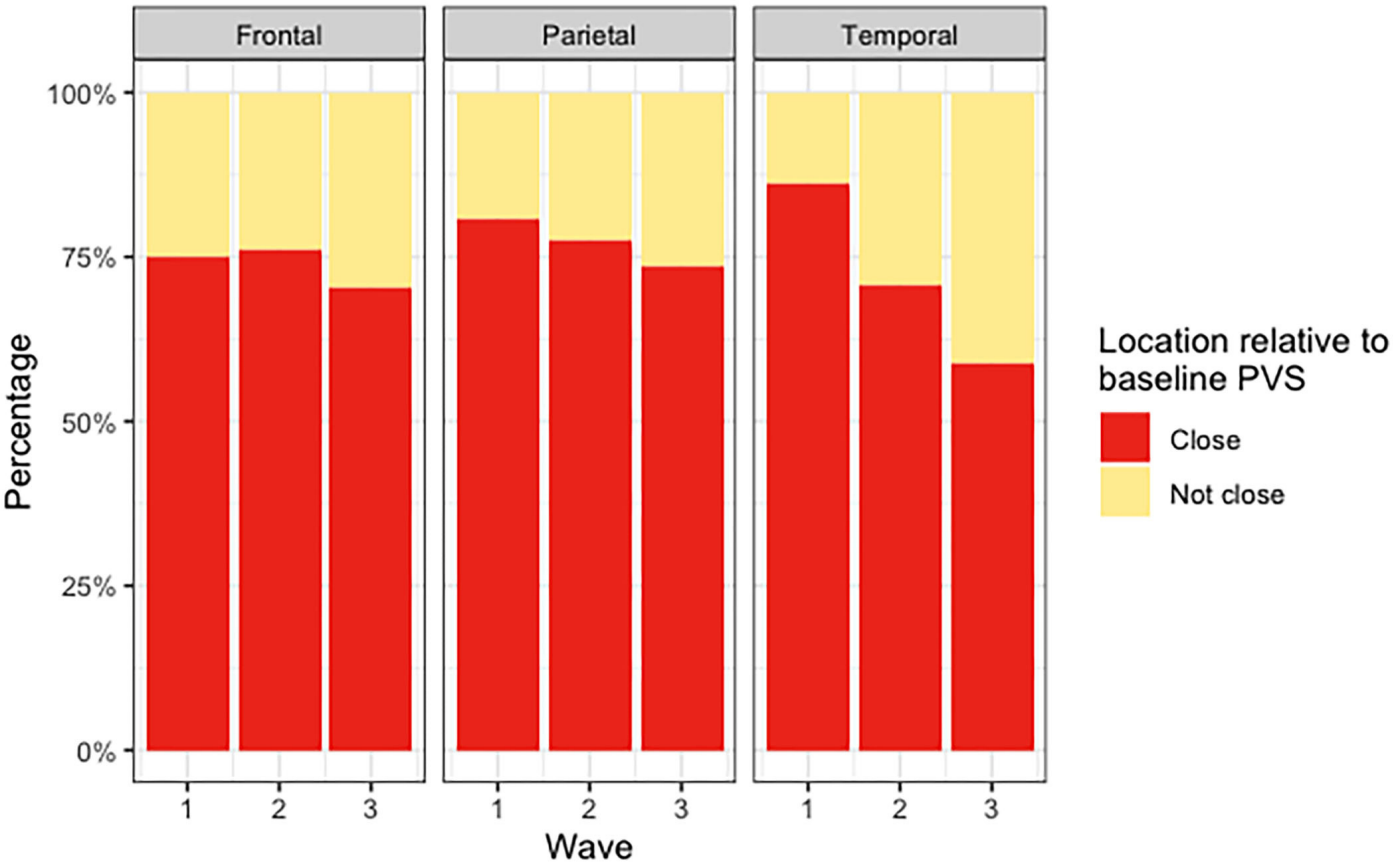
Percentages of deep WMH in waves 1, 2, and 3 that were found close and not close to baseline PVS in the frontal, parietal, and temporal regions.

The median count of deep WMH clusters “close” to baseline PVS (i.e., PVS identified at the first scanning wave) was greater than the median count of those “not close” at all time points (Figure 5). The counts were not normally distributed in this sample, with several participants having zero or very low counts of deep WMH clusters (see individual data in Supplementary files). A higher percentage of WMH clusters in wave 1 were found close to PVS (Table 2). In the rest of the waves, this pattern was also observed (wave 1: 77% “close,” 23% “not close”; wave 2: 76% “close,” 24% “not close”; wave 3: *n* = 69% “close,” 31% “not close”; Figure 6, Table 2). Despite far fewer deep WMH clusters being counted in the parietal and temporal regions than in the frontal region (Table 2), the percentages of these that were found “close” to baseline PVS (i.e., PVS identified at the first wave) were higher than those found “not close” in these regions (Figure 7; e.g., wave 1—frontal: 75% “close,” 25% “not close”; parietal: 81% “close,” 19% “not close”; temporal: 86% “close,” 14% “not close”). The occipital region was not included in Figure 7 as only one deep WMH cluster was counted in wave 1.

**Table 2:** Total number of deep WMH counted across the sample in waves 1, 2, and 3 that were found “close” and “not close” to baseline PVS, per lobar region and overall.

	**Close (%)**	**Not close (%)**	**Total (%)**
**Wave 1**
Frontal	343 (54.88)	114 (18.24)	457 (73.12)
Parietal	109 (17.44)	26 (4.16)	135 (21.60)
Temporal	25 (4.00)	4 (0.64)	29 (4.64)
Occipital	1 (0.16)	0 (0.00)	1 (0.16)
Overall	479 (76.64)	146 (23.36)	625 (100.00)
**Wave 2**
Frontal	401 (51.87)	125 (16.17)	526 (68.05)
Parietal	146 (18.89)	42 (5.43)	188 (24.32)
Temporal	34 (4.40)	14 (1.81)	48 (6.21)
Occipital	3 (0.39)	5 (0.65)	8 (1.03)
Overall	585 (75.68)	188 (24.32)	773 (100.00)
**Wave 3**
Frontal	633 (48.14)	264 (20.08)	897 (68.21)
Parietal	206 (15.66)	74 (5.63)	280 (21.29)
Temporal	60 (4.56)	42 (0.03)	102 (7.76)
Occipital	10 (0.76)	22 (1.67)	32 (2.43)
Overall	909 (69.12)	406 (30.87)	1,315 (100.00)

### Associations between progression of deep WMH clusters and proximity to PVS at wave 1

Between waves 1 and 2, the number of deep WMH clusters changed in 28 participants. There was a median increase of 2 in deep WMH clusters spatially close to baseline PVS (i.e., PVS detected at wave 1) and 1 in those not close. Between waves 2 and 3, the number of deep WMH clusters changed in all 29 participants. The median change in number spatially close to a PVS increased in 10, while the median change for those “not close” increased in 6. Logistic regression models with random effects found no significant associations between progression (defined by an increase in number) of deep WMH clusters and proximity of these clusters to PVS (i.e., either “close” or “not close”) at wave 1. Table 3 shows the odds ratios, 95% confidence intervals, and *p*-values for all models performed.

**Table 3:** Association between proximity of deep WMH to PVS and deep WMH progression by binary logistic regression with random effects.

**OR**
**(95% CI; *p*-value)**
	**Unadjusted**	**Adjusted for sex and VRF**
**Wave 1–2**
Not close	1.00	1.00
Close	1.93 (0.58–6.83; *p* = 0.28)	2.44 (0.68–8.82; *p* = 0.17)
**Wave 2–3**
Not close	1.00	1.00
Close	4.05 (0.09–176.00; *p* = 0.47)	2.00 (0.16–25.10; *p* = 0.59)

OR, odds ratio; 95% CI, 95% confidence interval; VRF, vascular risk factors.

### Longitudinal topological relationships

**Figure 8:**
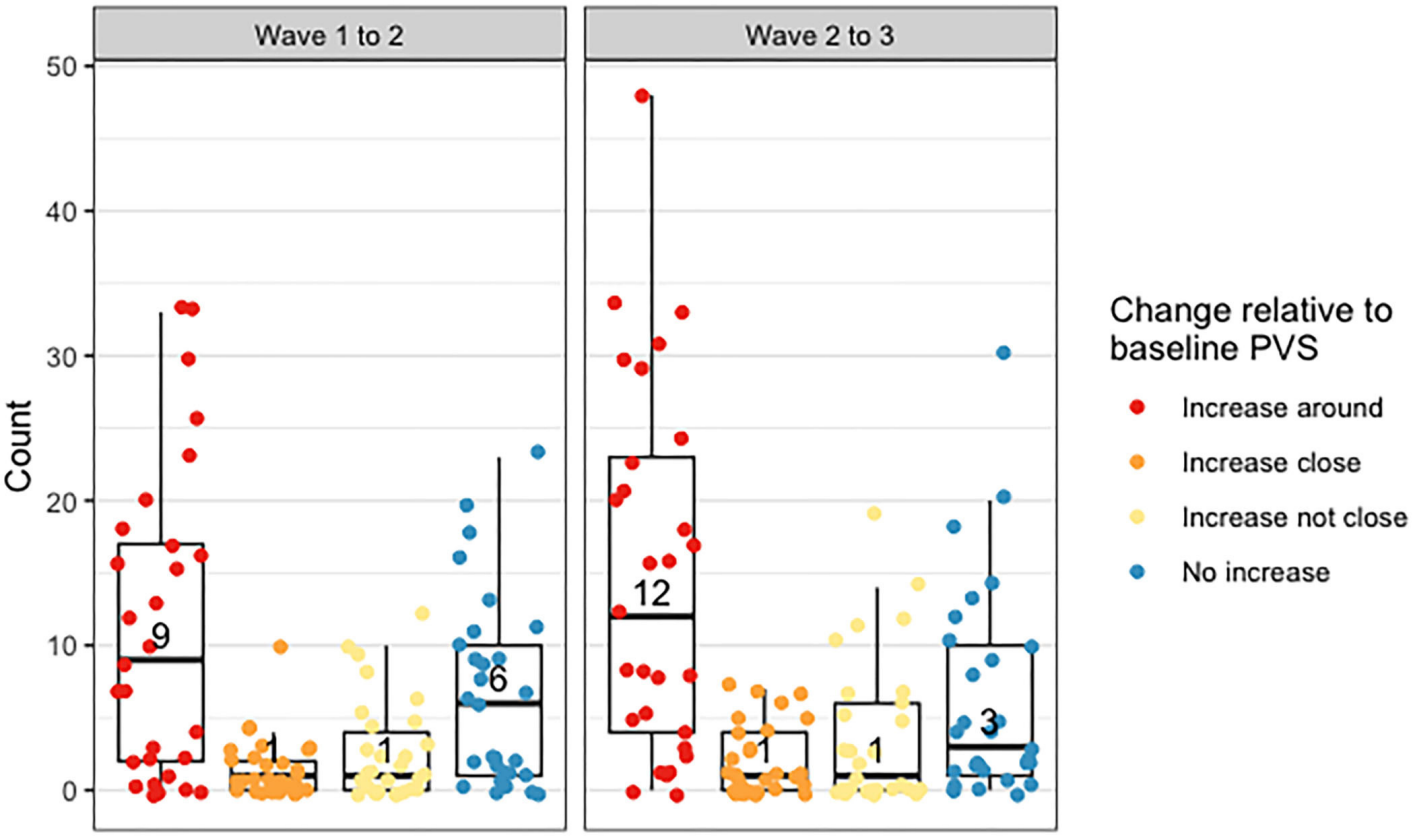
Distribution of counts of deep WMH that increased around, close and not close to baseline PVS, and did not increase between waves 1 and 2 and waves 2 and 3. The points plotted on each boxplot represent a different participant in the sample, and the numbers represent the median count.

**Figure 9:**
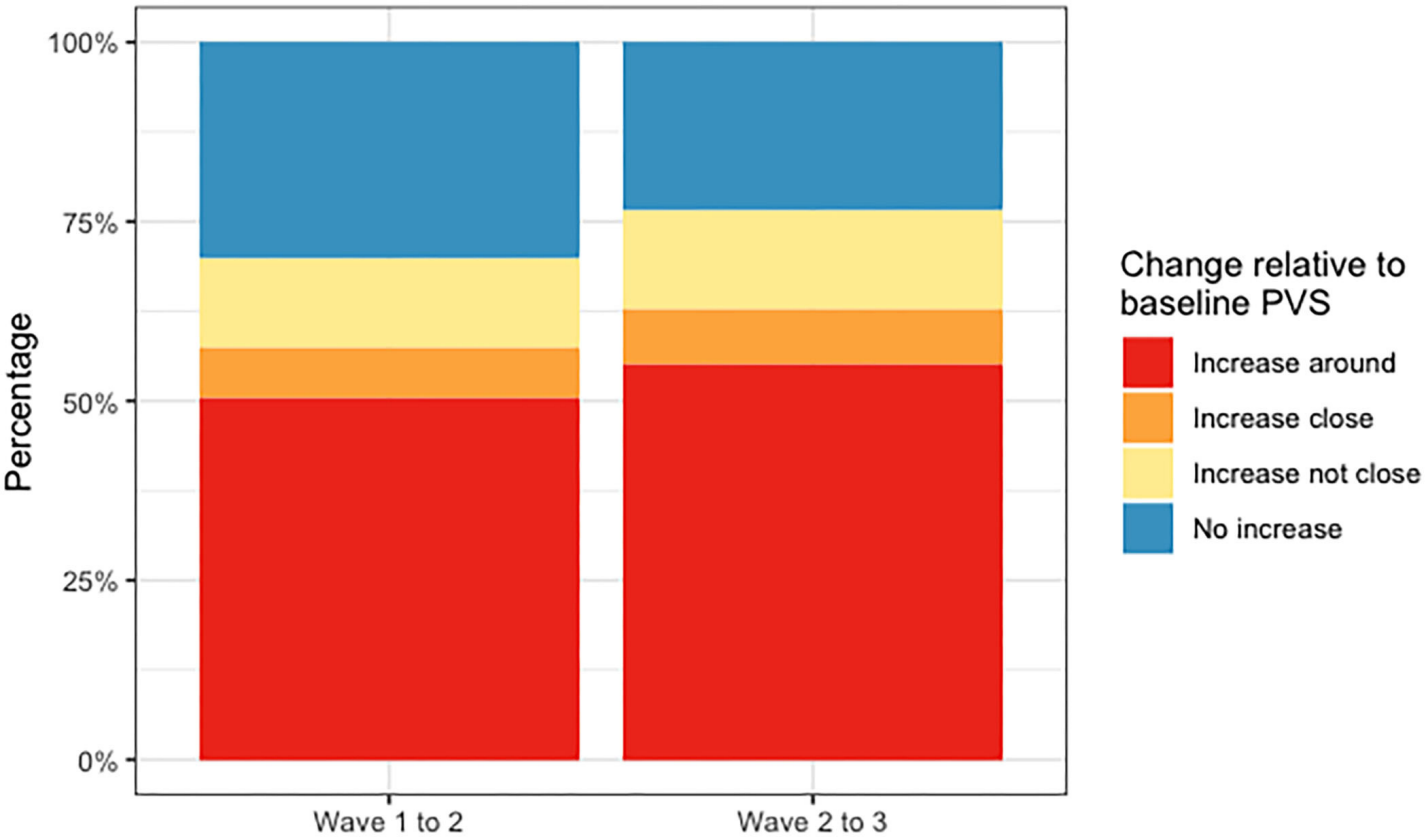
Percentages of deep WMH that increased around, close and not close to baseline PVS, and did not increase between waves 1 and 2 and waves 2 and 3.

**Figure 10:**
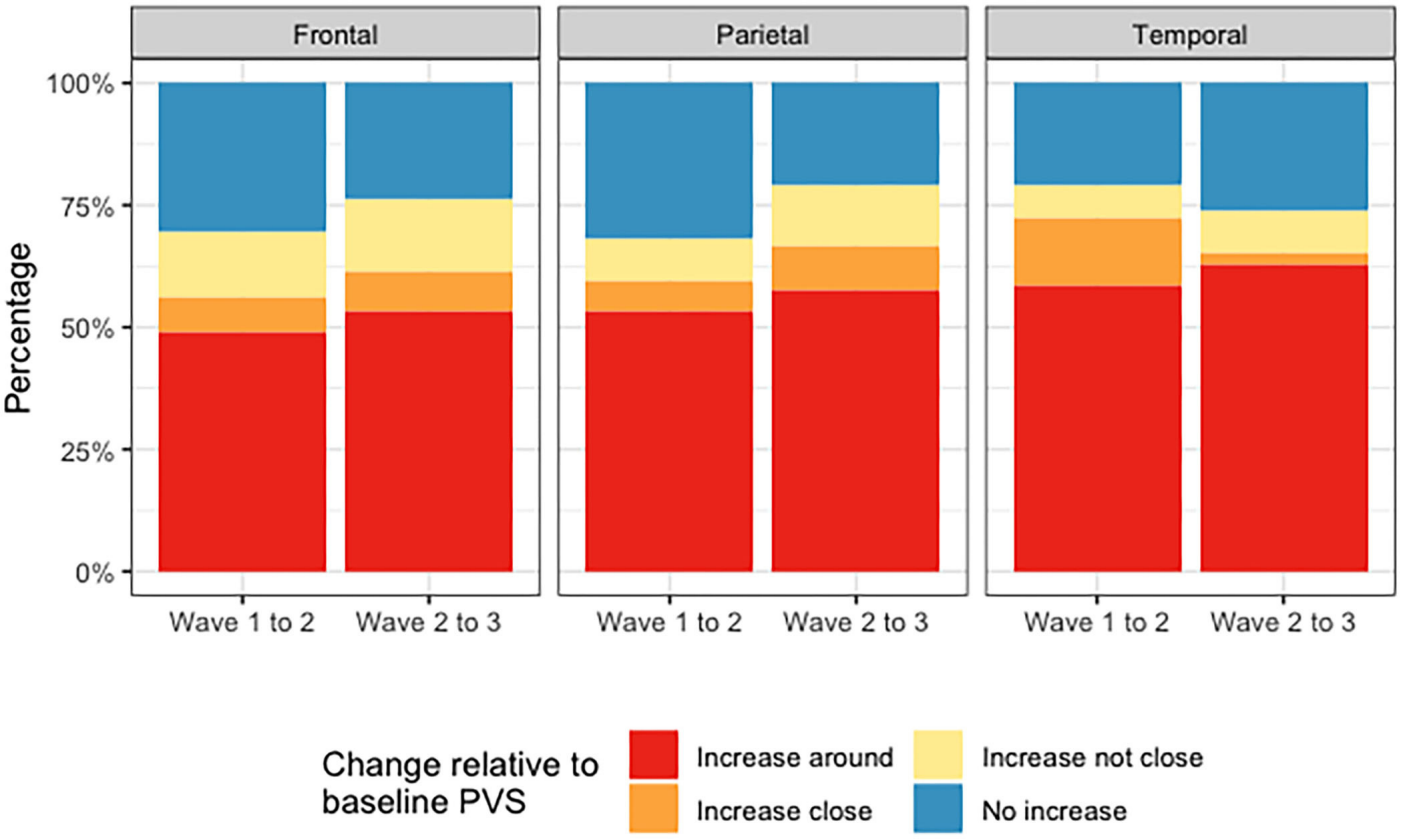
Percentages of deep WMH that increased around, close and not close to baseline PVS, and did not increase in the frontal, parietal, and temporal regions between waves 1 and 2 and waves 2 and 3.

Figure 8 shows the counts of changes in deep WMH clusters' morphology in relation to baseline PVS. The percentages of the different changes between waves 1 and 2 and waves 2 and 3 are displayed in Figure 9 and Table 4. A greater percentage of deep WMH clusters increased in size (waves 1–2: 70% increased and 30% did not increase; waves 2–3: 77% increased and 23% did not increase). Of those that increased, the most frequent change was an increase in size around the location of the already existent (i.e., “baseline”) PVS (waves 1–2: 73% “increased around,” 10% “increased close,” 17% “increased not close”; waves 2–3: 72% “increased around,” 10% “increased close,” 18% “increased not close”). When lobar regions were analyzed separately, these trends were also observed (Figure 10, Table 4). The occipital region was not included in Figure 10 because, as previously referred, only one WMH cluster was identified in this region at wave 1.

**Table 4:** Total number and percentages of deep WMH counted across the sample that increased around, close and not close to baseline PVS, and number of those that did not increase between waves 1 and 2 and waves 2 and 3, per region and overall.

	**Increase around (%)**	**Increase close (%)**	**Increase not near (%)**	**No increase (%)**	**Total (%)**
**Wave 1–2**
Frontal	227 (36.09)	33 (5.25)	62 (9.86)	139 (22.10)	461 (73.29)
Parietal	74 (11.76)	8 (1.27)	12 (1.91)	44 (6.99)	138 (21.94)
Temporal	17 (2.70)	4 (0.64)	2 (0.32)	6 (0.95)	29 (4.61)
Occipital	1 (0.16)	0 (0.00)	0 (0.00)	0 (0.00)	1 (0.16)
Overall	319 (50.71)	45 (7.15)	76 (12.08)	189 (30.05)	629 (100.00)
**Wave 2–3**
Frontal	283 (36.66)	42 (5.44)	79 (10.23)	124 (16.06)	528 (68.39)
Parietal	110 (14.25)	17 (2.20)	23 (2.98)	40 (5.18)	190 (24.61)
Temporal	29 (3.76)	1 (0.13)	4 (0.52)	12 (1.55)	46 (5.96)
Occipital	3 (0.39)	0 (0.00)	2 (0.26)	3 (0.39)	8 (1.04)
Overall	425 (55.05)	60 (7.77)	108 (13.99)	179 (23.18)	772 (100.00)

**Figure 11:**
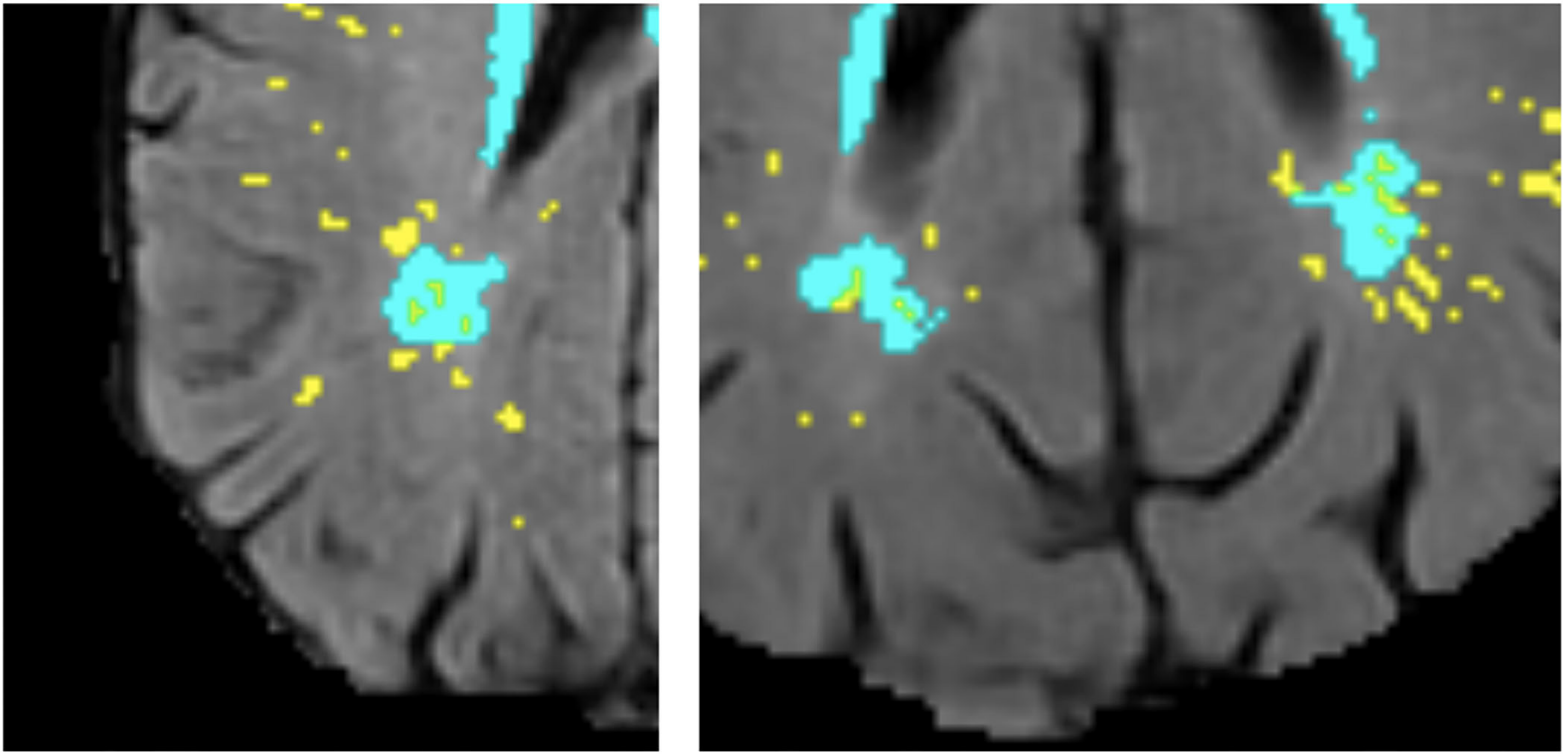
Examples of large deep WMH in the parietal region that were found spatially close to multiple PVS (segmentation masks for WMH in cyan and PVS in yellow, overlaid on FLAIR MRI).

Although far fewer deep WMH clusters were counted in the parietal and temporal regions compared with the frontal region, we more frequently observed larger WMH clusters forming around multiple PVS in the parietal and posterior temporal regions (Figure 11). Deep WMH clusters in the frontal region were typically smaller in volume with a small area of continuity with a PVS.

### Influence of the PVS burden and WMH cluster size in the spatial relationships found

The PVS volume and mean size did not seem to have influenced the spatial relationships found. As shown in the Supplementary data spreadsheet “PVSandWMHburden_analysis.xlsx,” the *R*^2^ value for the linear regression between number of CSO PVS “close” to WMH and WMH volume for the scanning wave 1 was 0.484, for wave 2 it was 0.421, and for wave 3 it was 0.542. On the contrary, the *R*^2^ value for the linear regression between CSO PVS volume and WMH volume for wave 1 was 0.0539, and between mean CSO PVS size and WMH volume, it was 0.049, thus indicating that approximately half of the variation in WMH volume at each wave can be rather explained by the number of PVS “close” to WMH instead of by the PVS burden. Similar results were obtained while evaluating the univariate association between the number of PVS considered “close” or “around” WMH that increased from waves 1 to 2 (*R*^2^ = 0.581) and from waves 2 to 3 (*R*^2^ = 0.512), and the WMH volume at waves 2 and 3, respectively. Paired t-test analysis of the density of PVS “close” vs. “not close” showed significant differences between both variables at all waves [wave 1: mean (SD) PVS “close” = 0.114 (0.103) ml^−1^, mean (SD) PVS “not close” = 0.0349 (0.0406) ml^−1^, *p* = 1.259 × 10^−6^; wave 2: mean (SD) PVS “close” = 0.140 (0.117) ml^−1^, mean (SD) PVS “not close” = 0.0450 (0.0560) ml^−1^, *p* = 2.443 × 10^−7^; wave 3: mean (SD) PVS “close” = 0.216 (0.163) ml^−1^, mean (SD) PVS “not close” = 0.100 (0.113) ml^−1^, *p* = 1.133 × 10^−8^).

To classify the WMH clusters into “big” or “small” while avoiding inter-/intra-observer bias, we looked at the deep Fazekas scores (provided as part of the Supplementary data) given by the neuroradiologist. As defined in Fazekas et al. (49), the deep WMH scores are equal to 1 if small punctate and very few, 2 if bigger and in higher number, and 3 if large and confluent with the periventricular WMH. From the whole sample, only one subject (subject no. 16) has deep Fazekas scores equal to 3 and two subjects (subject nos. 7 and 11) have deep Fazekas scores equal to 2. Therefore, it is unlikely that the spatial relationship between WMH and PVS would have been driven by the size of the WMH clusters.

## Discussion

This pilot study in a sample of participants from a community-dwelling Scottish cohort is the first longitudinal study to date that evaluated the topological spatial relationship between PVS and deep WMH clusters. By carefully analyzing data across six years acquired in three equally spaced time points, our study provides further insights into this novel area of research by corroborating the hypothesis that some deep WMH may increase in size and form around PVS, previously inferred from cross-sectional data. Our results, therefore, suggest that in normal aging, WMH formation may be linked to impaired brain clearance mechanisms in addition to the vascular origin referred to in the current literature (42). From our observations, it is possible to infer that worsening interstitial fluid drainage over time can cause further accumulation of fluid in the white matter immediately adjacent to where drainage *via* PVS was previously impaired. This is consistent with the proposed mediating role of free water in the brain in the association seen between PVS and deep WMH clusters by a previous cross-sectional study (26).

Moreover, our results indicate that the spatial relationships found are not likely to be artificially driven by the PVS volume and mean size or by the size of WMH clusters. Clinical reviews have previously proposed that WMH may preferentially form around PVS (5, 42). Another review that examined the pathological evidence for the failure of the brain clearance mechanisms as a significant cause of the overall pathology found particularly in the aging brain, stated that enlarged PVS reflect impaired interstitial fluid drainage and lead to the development of WMH (43). Our study for the first time provides evidence to support this claim, by analyzing the spatial proximity, distribution, and evolution of WMH in relation to PVS in brain scans from an age-homogeneous cohort representative of normal aging across 6 years within the eight decade of life.

Previously published data on topological relationships observed a marked difference between the percentages of WMH clusters spatially connected and not connected to PVS (26) when compared with those of our study. However, this may be due to several differences in study design. The age of the sample utilized by Huang et al. (26) was younger, with an inter-quartile range of 56–65 years, compared with that of our sample, where participants were aged ~72, 75, and 78 years when relationships were analyzed. As both PVS and WMH are independently correlated with increasing age (44, 45), this may have influenced the differences seen. In terms of vascular disease, our sample is also more heterogeneous as it includes individuals with cardiovascular disease and total Fazekas scores ranging from 1 to 5. Also, different from our study, Huang et al. analyzed a random sample of deep WMH clusters rather than all present. While this was more feasible in a larger sample and allowed better appreciation of relationships in 3D, it may have introduced within-subject sampling error.

Although our longitudinal analysis revealed an increase in size of the WMH clusters close to or overlapping with the PVS identified at the first wave of scanning, our statistical analysis suggests that the number of deep WMH clusters spatially close to PVS was no more likely to increase than those spatially not close and that the location of deep WMH clusters in relation to PVS at the first scan was not a predictor for WMH progression in this sample. There are several possible reasons why no statistically significant associations were found. The baseline scan, obtained at a mean age close to 72 years, already showed most of the WMH clusters being located close to PVS, and most of these clusters experienced a growth over the periods evaluated. Therefore, it is reasonable that they would appear around or close to PVS at follow-up scans without necessarily meaning that the proximity to a PVS was related to their enlargement. The homogeneity of the sample in terms of age compensated the likelihood of biasedness of the adjusted statistical models, as age was not a factor needed to account for. Nevertheless, given the small size of the sample, the number of outcome events per predictor variable was slightly less than the recommended 10 (46). A Binary logistic regression with random effects was chosen because the alternatives were deemed beyond the scope of this study due to the complexity and number of assessments, which such a small sample size would be unlikely to support. A larger study would use more sophisticated analysis methods, such as Poisson's regression, to fully exploit the count nature of the data (47). Defining deep WMH clusters' progression as an increase in their number for statistical analyses was a further limitation as it did not take into account change in volume of individual clusters and the possibility of multiple clusters coalescing into one over time, which was observed in some cases. For such a detailed analysis to be performed computationally, it would have required access to a large “ground-truth” databank currently non-existent. Also, the analysis of the odds of deep WMH clusters close to PVS increasing in number to those not close to PVS is complex as it is unclear what deep WMH clusters not close to PVS represent. Although their appearance is unlikely to be related to the consequences of impaired interstitial fluid drainage, they may be just as likely to increase in number for different reasons.

Another limitation of our study is the relatively low spatial resolution of the images for the assessment of these types of structures (i.e., 1 × 1 × 2 mm^3^ at 1.5 tesla), which, despite resampling, may have introduced an error in counting the PVS that occur parallel to the axial plane. Relying on segmentation to define the relationships seen, despite improving the reliability and reproducibility in the assessments, meant that accuracy of the data collected was dependent on the accuracy of segmentation, particularly for PVS for which manual edit of each of these small features individually is impractical and highly prone to individual observer's considerations, especially for cases with a high burden of them. Inaccuracies in the segmentation of baseline PVS in this cohort as a whole have been recognized (23), but the sample analyzed was double-checked visually to ensure quality in the analyses. The reliance in the segmentation could have also meant that small increase (or decrease) in the number of voxels (e.g., one or two voxels) recognized by the algorithm could have been considered an actual biological change. The filtering method used in the PVS segmentation is widely recognized for its robustness, and given the image normalization steps also conducted, an artificial increase in the number of connected voxels considered PVS is not highly likely. The change in WMH volume, however, could have been artificially generated by the algorithms, especially given the undefined borders of some lesions and the differences in defining the thresholds by the two methods used to segment them. To overcome this limitation, the change in the WMH cluster size was evaluated visually and considered as such only if this was perceived nearly as two times (or half) or more the original cluster size. Also, it is not clear whether punctate WMH that pathology studies recognize as WMH composed of and characterized by enlarged PVS (48–50) are segmented as PVS, WMH, or both. This may have also affected how accurately deep WMH clusters were classified. Lastly, due to the visual and 3D nature of the assessments, despite the high intra-observer reliability, one cannot discard the possibility of potentially mistaken classification of WMH clusters close to PVS diagonally across slices, or double counting of the same cluster in different planes. Once the segmentations are done, distance from each cluster to PVS masks could have been calculated computationally, for example, using the Euclidean or Manhattan distances, but anyway they would have needed to be checked visually, because to avoid considering PVS proximities to more than one WMH cluster, arbitrary limits would have needed to be imposed, complicating the interpretability of the results.

The fact that only one observer performed the visual assessments limited the sources of variation in the dataset. Counting all deep WMH clusters is very time-consuming, especially in participants with a high WMH burden, so the methodology used here would not be feasible in large samples. Nonetheless, it proved a useful way of obtaining data on topological relationships between deep WMH clusters and PVS, both cross-sectionally and longitudinally, to test our hypotheses. Due to the novelty of this research, there are no reliably annotated data to train any machine learning algorithm to reliably classify large data fully automatically across the whole brain. Existent automatic classifiers that demand less or no data at all have a suboptimal accuracy for being applied in clinical studies or test clinically relevant hypotheses. Therefore, future research should continue to use the methodology developed for this study to generate ground-truth data in a larger sample, reassess the relationships seen, and re-evaluate the utility of the presented paradigm to determine its usefulness in predicting WMH progression. Moreover, future studies should include in the analyses participants with history of previous strokes to investigate differences in tendencies over time (if they exist) between individuals who had a stroke against those who did not. It will be also useful to validate the results against the percentage overlap between segmentation masks for PVS and WMH, which would better take into account differences in volume. As described in Materials and Methods section and in previous publications from this cohort, WMH binary masks separately for periventricular and deep regions are not currently available. Therefore, with the data available at present such computational analysis would have been misleading.

As our findings support the idea that PVS enlargement (i.e., for PVS to be visible in MRI) may precede WMH development, future research on better understanding what causes PVS enlargement would be important. MRI-visible PVS in the centrum semiovale are linked to amyloid deposition (51, 52); therefore, ways to prevent this would be clinically beneficial.

## Conclusion

In this pilot study, more deep WMH clusters were found spatially close to a baseline PVS than distant, and more than half progressed with time, increasing around a baseline PVS. Although this sample is very small, these findings support a mechanistic link between these two cSVD features that may improve our understanding of the mechanisms involved in cSVD and WMH development to help reduce and prevent associated symptoms and neurological conditions. Formal statistical comparisons of severity of these two SVD markers yielded no associations between deep WMH clusters progression and the location of these clusters relative to PVS. The sample size should be increased to confirm these associations. Future research should also explore more feasible ways of analyzing these relationships (i.e., automatically) and the causes of PVS enlargement to continue furthering our understanding of the mechanisms involved in cSVD.

## Data availability statement

The original contributions presented in the study are included in the article/Supplementary material, further inquiries can be directed to the corresponding author.

## Ethics statement

The studies involving human participants were reviewed and approved by Lothian (REC 07/MRE00/58) and Scottish Multicentre (MREC/01/0/56) Research Ethics Committees. The patients/participants provided their written informed consent to participate in this study.

## Author contributions

LB, MV, JW, and FC were involved in conceptualization. AB, LB, MV, SM, RM, EB, and MS were involved in data curation. AB was involved in formal analysis and visualization. SC, MB, ID, and JW were involved in funding acquisition. AB, LB, JW, and FC were involved in investigation. AB, LB, RD, MV, FC, and JW contributed to methodology. LB, RB, RM, and JW were involved in project administration. LB, JW, SC, ID, and MB contributed to supervision and resources. LB, RD, MV, and FC contributed to software. AB, MV, and LB were involved in validation. AB and MV were involved in writing—original draft preparation. All authors were involved in writing—review and editing. All authors contributed to the article and approved the submitted version.

## Funding

This study was partially funded by the Selfridges Group Foundation under the Novel Biomarkers 2019 scheme (ref UB190097) administered by the Weston Brain Institute. The LBC1936 is supported by Age UK as The Disconnected Mind Project (http://www.disconnectedmind.ed.ac.uk), the Medical Research Council [G1001245/96099] and The University of Edinburgh. LBC1936 MRI brain imaging was supported by Medical Research Council (MRC) grants [G0701120], [G1001245], [MR/M013111/1], and [MR/R024065/1]. Magnetic Resonance Image acquisition and analyses were conducted at the Brain Research Imaging Centre, Neuroimaging Sciences, University of Edinburgh (www.bric.ed.ac.uk) which is part of SINAPSE (Scottish Imaging Network—A Platform for Scientific Excellence) collaboration (www.sinapse.ac.uk) funded by the Scottish Funding Council and the Chief Scientist Office. This work was supported by the Centre for Cognitive Ageing and Cognitive Epidemiology, funded by the Medical Research Council and the Biotechnology and Biological Sciences Research Council (MR/K026992/1), the Row Fogo Charitable Trust (BRO-D.FID3668413), the European Union Horizon 2020, (PHC-03-15, project No 666881), SVDs@Target, the Fondation Leducq Transatlantic Network of Excellence for the Study of Perivascular Spaces in Small Vessel Disease, ref no. 16 CVD 05, the US National Institutes of Health (R01AG054628), a Sir Henry Dale Fellowship jointly funded by the Wellcome Trust, the Royal Society (SRC, Grant Number 221890/Z/20/Z), and the Medical Research Council UK Dementia Research Institute at the University of Edinburgh. None of the funders have any role in the collection and processing of the data or the content presented in this manuscript.

## Conflict of interest

The authors declare that the research was conducted in the absence of any commercial or financial relationships that could be construed as a potential conflict of interest.

## Publisher's note

All claims expressed in this article are solely those of the authors and do not necessarily represent those of their affiliated organizations, or those of the publisher, the editors and the reviewers. Any product that may be evaluated in this article, or claim that may be made by its manufacturer, is not guaranteed or endorsed by the publisher.
