# Supplementary figures and images for: Topological relationships between perivascular spaces and progression of white matter hyperintensities: A pilot study in a sample of the Lothian Birth Cohort 1936
Source: Front Neurol. 2022 Aug 24;13:889884. doi: 10.3389/fneur.2022.889884 (PMC9449650; doi:10.3389/fneur.2022.889884)

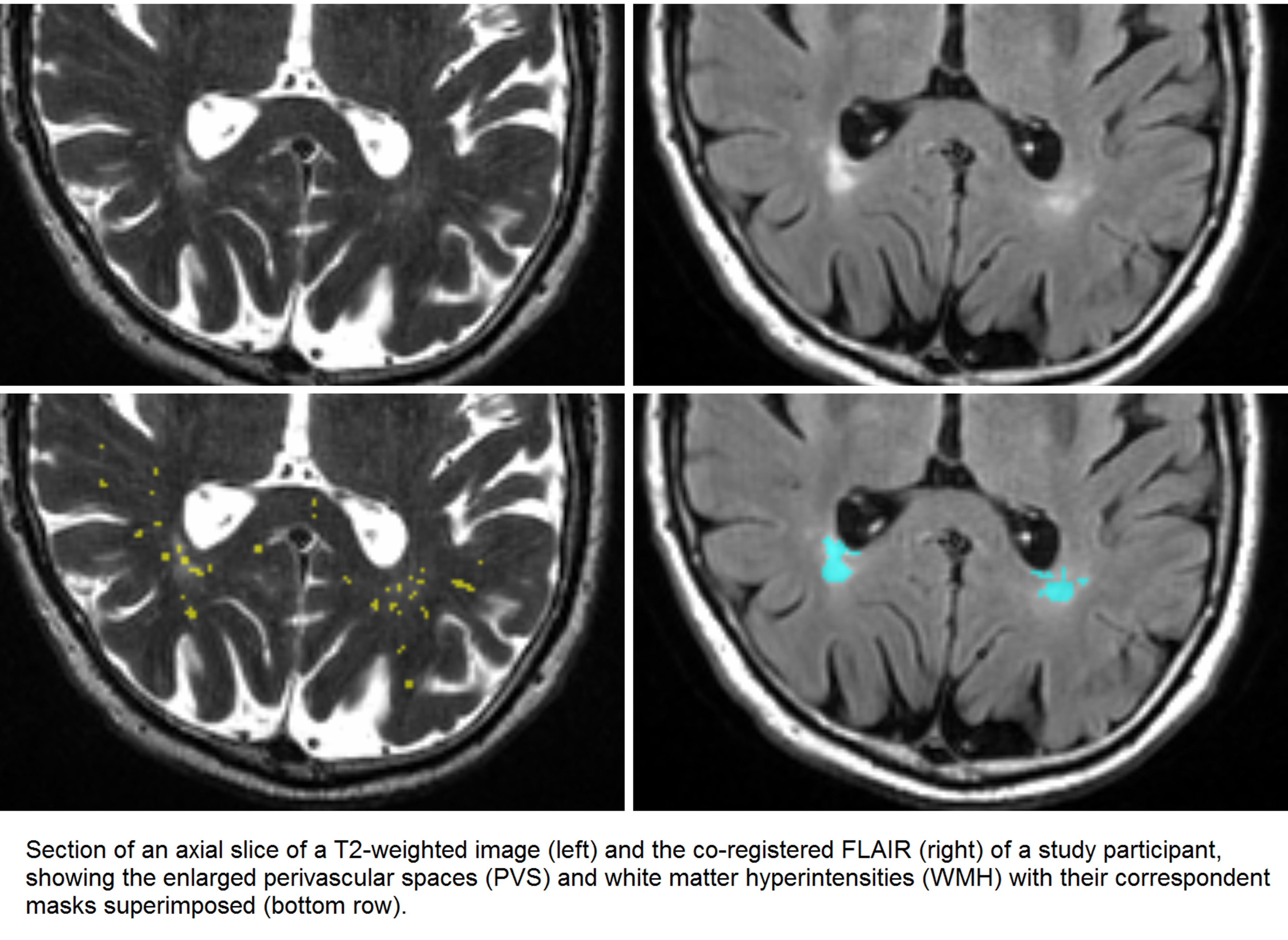

Supplement: Supplementary file 1 [file Image_1.TIF]

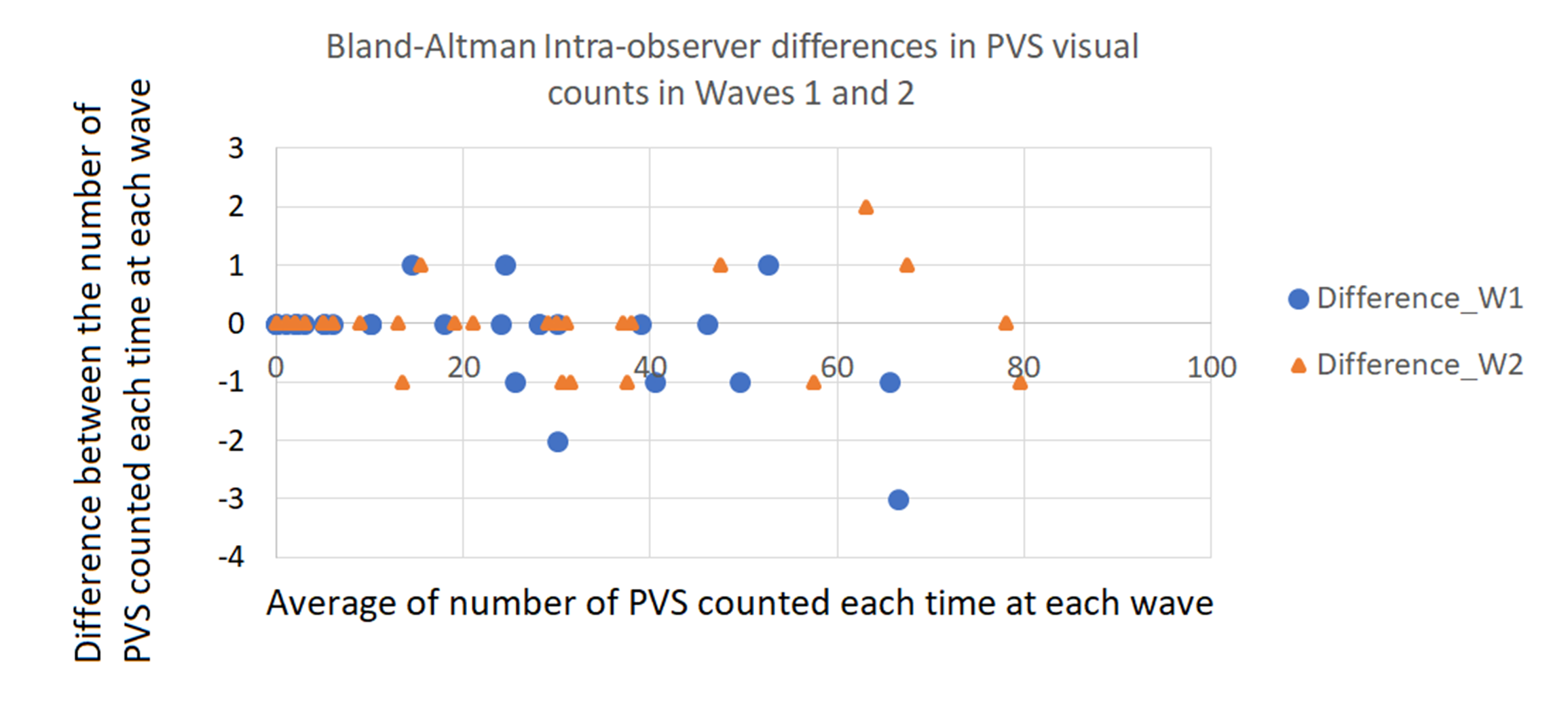

Supplement: Supplementary file 2 [file Image_2.TIF]
